# The nascent polypeptide in the 60S subunit determines the Rqc2-dependency of ribosomal quality control

**DOI:** 10.1093/nar/gkab005

**Published:** 2021-01-28

**Authors:** Masato Mizuno, Shuhei Ebine, Okuto Shounai, Shizuka Nakajima, Shota Tomomatsu, Ken Ikeuchi, Yoshitaka Matsuo, Toshifumi Inada

**Affiliations:** Graduate School of Pharmaceutical Science, Tohoku University, Aoba-ku, Sendai 980-8578, Japan; Graduate School of Pharmaceutical Science, Tohoku University, Aoba-ku, Sendai 980-8578, Japan; Graduate School of Pharmaceutical Science, Tohoku University, Aoba-ku, Sendai 980-8578, Japan; Graduate School of Pharmaceutical Science, Tohoku University, Aoba-ku, Sendai 980-8578, Japan; Graduate School of Pharmaceutical Science, Tohoku University, Aoba-ku, Sendai 980-8578, Japan; Graduate School of Pharmaceutical Science, Tohoku University, Aoba-ku, Sendai 980-8578, Japan; Graduate School of Pharmaceutical Science, Tohoku University, Aoba-ku, Sendai 980-8578, Japan; Graduate School of Pharmaceutical Science, Tohoku University, Aoba-ku, Sendai 980-8578, Japan

## Abstract

Ribosome stalling at tandem CGA codons or poly(A) sequences activates quality controls for nascent polypeptides including ribosome-associated quality control (RQC) and no-go mRNA decay (NGD). In RQC pathway, Hel2-dependent uS10 ubiquitination and the RQC-trigger (RQT) complex are essential for subunit dissociation, and Ltn1-dependent ubiquitination of peptidyl-tRNA in the 60S subunit requires Rqc2. Here, we report that polytryptophan sequences induce Rqc2-independent RQC. More than 11 consecutive tryptophan residues induced RQC in a manner dependent on Hel2-mediated ribosome ubiquitination and the RQT complex. Polytryptophan sequence-mediated RQC was not coupled with CAT-tailing, and Rqc2 was not required for Ltn1-dependent degradation of the arrest products. Eight consecutive tryptophan residues located at the region proximal to the peptidyl transferase center in the ribosome tunnel inhibited CAT-tailing by tandem CGA codons. Polytryptophan sequences also induced Hel2-mediated canonical RQC-coupled NGD and RQC-uncoupled NGD outside the stalled ribosomes. We propose that poly-tryptophan sequences induce Rqc2-independent RQC, suggesting that CAT-tailing in the 60S subunit could be modulated by the polypeptide in the ribosome exit tunnel.

## INTRODUCTION

Cells have various quality control systems that selectively degrade aberrant mRNA and defective proteins to ensure precise expression of genome-encoded information. Ribosome stalling during elongation results in degradation of both the mRNA and the arrested nascent protein by mRNA surveillance pathways and the ribosome-associated quality control (RQC) systems (1–4). In yeast, the RQC pathway is triggered by ubiquitination of the ribosomal protein uS10 at specific lysine residues by the E3 ubiquitin ligase Hel2 (ZNF598 in mammals) and the E2 enzyme Ubc4 (5). Activation of the RQC pathway requires an additional protein complex, the RQC-trigger (RQT) complex (5). It is composed of the RNA helicase-family protein Slh1, the ubiquitin-binding protein Cue3 and yKR023W/Rqt4 (5,6). The RQT complex is associated with the ribosome and Hel2, and the ubiquitin-binding activity of Cue3/Rqt3 and the ATPase activity of Slh1 are crucial for triggering RQC (5). Recent studies in the mammalian system demonstrated that ZNF598 ubiquitinates the ribosomal proteins eS10 at K138 and K139, and uS10 at K4 and K8. This ubiquitination triggers the RQC response on ribosomes stalled on a polylysine-encoding mRNA reporter, indicating that the role of ribosome ubiquitination in quality control is conserved (7–9). The colliding ribosome was identified as a substrate for ribosome ubiquitination by Hel2/ZNF598 (10,11). *SDD1* an endogenous RQC substrate, was translated *in vitro* to reconstitute Hel2-dependent polyubiquitination of collided trisomes (12). Cryo-EM revealed a distinct trisome architecture, a structure that enables more efficient recognition by Hel2 than that of disomes. This promotes the dissociation of the first stalled polyubiquitinated ribosome by the Slh1 helicase subunit of the RQT complex in an ATP-dependent manner (12). The human RQC-trigger (hRQT) complex, an ortholog of the yeast RQT complex, plays crucial roles in RQC (13,14). The hRQT complex is composed of ASCC3, ASCC2 and TRIP4, which are orthologs of the RNA helicase Slh1, ubiquitin-binding protein Cue3 and zinc-finger type protein Rqt4, respectively. The ATPase activity of ASCC3 and the ubiquitin-binding activity of ASCC2 are crucial for triggering RQC (13,14), and the hRQT complex recognises the ubiquitinated stalled ribosome and induces subunit dissociation to facilitate RQC (13,14).

Stalled ribosomes are ubiquitinated and dissociated into subunits, yielding 60S ribosome-nascent polypeptide complexes (RNCs) (5–15). The ubiquitin ligase Ltn1 (Listerin in mammals) plays a crucial role in RQC (4,15,16). A forward genetics approach in a mouse model was used to show that mutation of the gene encoding Ltn1/Listerin leads to neurodegeneration (17). Rqc2 (NEMF in mammals) interacts with 60S RNCs and recruits Ltn1/Listerin, which ubiquitinates peptidyl-tRNAs on dissociated 60S subunits (3,4). In the 60S subunit, the Rqc2 protein catalyses the C-terminal extension of stalled tRNA-bound peptides with alanine and threonine residues (CAT-tails) in a non-canonical mRNA-independent elongation reaction (18,19). CAT-tailing enables the degradation of substrates that lack a Ltn1p-accessible ubiquitination site by exposing a lysine residue that is normally sequestered in the ribosome exit tunnel (19). In the context of nascent chain degradation in budding yeast, CAT-tailing is a fail-safe mechanism that expands the range of RQC-degradable substrates (19). Brandman et al. developed quantitative techniques to assess the effect of CAT-tails on the degradation of stalled polypeptides. The results showed that CAT-tails increase the efficiency of Ltn1 for targeting structured polypeptides, which are otherwise poor Ltn1 substrates, in *Saccharomyces cerevisiae* (20). In cases in which Ltn1 fails to ubiquitinate stalled polypeptides or becomes limiting, CAT-tails act as degrons, marking proteins for proteasomal degradation. CAT-tailing of nuclear-encoded mitochondrial proteins prevents aggregation of the mitochondrial proteome (21). Vms1 plays a crucial role in RQC by counteracting inhibitory CAT-tailing functions. Vms1 terminates CAT-tail formation during RQC by acting as a release factor via nuclease activities (22–26).

We previously reported that polytryptophan sequences also induce RQC (32). However, the mechanism by which the polytryptophan peptide induces translation arrest and quality control remains unclear. In this study, we demonstrate that polytryptophan sequences induce RQC and no-go mRNA decay (NGD), and elucidate the underlying mechanism. We identified >11 consecutive tryptophan residues inducing RQC, and we demonstrated that RQC factors such as Asc1, Hel2 and Slh1–Cue3–Rqt4 are essential for RQC. We show that the induction of RQC by polytryptophan sequences is not coupled with CAT-tailing. We found that eight consecutive tryptophan residues located at the discrimination gate of the ribosome tunnel inhibited CAT-tailing, suggesting that steric hindrance between the constriction site of the ribosome tunnel and the nascent polypeptides inhibits CAT-tailing in the 60S subunit. Rqc2 was not required for Ltn1-dependent degradation of the arrested products, indicating that polytryptophan sequences induce Rqc2-independent RQC. We propose that the properties of the nascent polypeptide in the 60S subunit determine the Rqc2-dependency of RQC.

## MATERIALS AND METHODS

### Yeast strains

Yeast strains are listed in Supplementary Table S1. Yeast *Saccharomyces cerevisiae* W303-1a-based strains were obtained by established recombination techniques using PCR-amplified cassette sequences (*kanMX4*, *hphMX4*, *natMX4*, *natNT2* or *HISMX6*) (12,15). To construct strains carrying essential ribosomal protein genes (uS10, uS3 and eS7AeS7B), the shuffle strain transformed with plasmids expressing mutant ribosomal protein products was grown on an SDC plate containing 0.5 mg/ml of 5-fluoro-orotic acid (5-FOA, #F9001-5, Zymo Research, Irvine, CA, USA), and URA3 absent strains were isolated.

### Plasmids

Plasmids used in this study are listed in Supplementary Table S2. DNA cloning was performed by PCR amplification with gene-specific primers using PrimeSTAR HS DNA polymerase (#R010A, Takara-bio, Shiga, Japan) and T4 DNA ligase (#M0202S, NEB, Ipswich, MA, USA). All cloned DNAs amplified by PCR were verified by sequencing.

### Yeast culture and media

Yeast cells were cultured with YPD or synthetic complete (SC) medium with 2% glucose at 30°C, and harvested at log phase by centrifugation after discarding the medium.Cell pellets were frozen in liquid nitrogen immediately after harvest and stored at –80°C until used.

### RNA isolation for determination

Total RNA solutions were prepared using the acidic phenol RNA extraction method as follows: the cell pellet was resuspended with 300 μl RNA buffer (300 mM NaCl, 20 mM Tris–HCl pH 7.5, 10 mM EDTA, 1% SDS, dissolved in DEPC-treated MilliQ water at room temperature) on ice, followed by immediate addition of 300 μl water-saturated phenol and vortex mixing for 10 s. The mixture was incubated at 65°C for 5 min, mixed by vortex for 10 s and chilled on ice for 5 min. After centrifugation at 16 000 × g for 5 min at room temperature, 300 μl of the water layer was transferred to a new 1.5 ml RNase-free tube. After addition of 250 μl water-saturated phenol, the procedure was repeated; 300 μl of the water layer was transferred to a new 1.5 ml RNase-free tube, and 300 μl water-saturated phenol/chloroform (1:1) was added, followed by a 10 s vortex and centrifugation at 16 000 × g for 5 min at room temperature. Then, 300 μl of the water layer was transferred to a new 1.5 ml RNase-free tube, and 300 μl water-saturated phenol/chloroform/isoamylalchol (25:24:1) was added, followed by a 10 s vortex and centrifugation at 16 000 × g for 5 min at room temperature. Then, 300 μl of the water layer was transferred to a new 1.5 ml RNase-free tube and subjected to ethanol precipitation. The RNA pellet was finally dissolved with 20–30 μl DEPC-treated water to obtain a total RNA solution.

### RNA electrophoresis and northern blotting

A volume of 6 μl (2.5 μg) total RNA solution was mixed with 24 μl glyoxal mix [600 μl DMSO, 200 μl deionised 40% glyoxal, 120 μl of 10 × MOPS buffer (200 mM MOPS, 50 mM NaOAc, 10 mM EDTA pH 7.0), 62.5 μl of 80% glycerol and 17.5 μl DEPC-treated water in 1 ml] and 3 μl RNA loading buffer (50% glycerol, 10 mM EDTA pH 8.0, 0.05% bromophenol blue and 0.05% xylene cyanol). The mixture was incubated at 74°C for 10 min, followed by incubation on ice for 10 min to obtain RNA samples. Aliquots of 25 μl of each sample were electrophoresed at 200 V for 40 min on a 1.2% or 2% agarose gel in 1× MOPS buffer (20 mM MOPS, 5 mM NaOAc, 1 mM EDTA pH 7.0), followed by transfer of RNA to Hybond-N+ membranes (GE healthcare, Chicago, IL, USA) with 20× SSC (3 M NaCl and 300 mM trisodium citrate dihydrate) for 18 h using a capillary system. RNA was cross-linked on the membrane using a CL-1000 ultraviolet crosslinker (UVP) at 120 mJ/cm^2^. The membrane was then incubated with DIG Easy Hyb Granules (#11796895001, Roche) for 1 h in a hybridization oven at 50°C. A DIG-labeled probe prepared using the PCR DIG Probe Synthesis Kit (#11636090910, Roche) was added and incubated for >18 h, followed by two washes with wash buffer I (2× SSC, 0.1% SDS) for 15 min in a hybridization oven at 50°C, and an additional wash with wash buffer II (0.1× SSC, 0.1% SDS) for 15 min at 50°C. The membrane was then washed with 1× maleic acid buffer (100 mM maleic acid, 150 mM NaCl pH 7.0, adjusted with NaOH) for 10 min at room temperature and incubated with Blocking Reagent (#11096176001, Roche) for 30 min. Anti-Digoxigenin-AP, Fab fragments (#11093274910, Roche) was added to the Blocking Reagent, and the membrane was further incubated for 1 h. After that, the membrane was washed with wash buffer III (1× maleic acid buffer, 0.3% Tween 20) for 10 min three times, and equilibrated with equilibration buffer (100 mM Tris–HCl, 100 mM NaCl pH 9.5). The membrane was reacted with CDP-star (#11759051001, Roche) for 10 min, and chemiluminescence was detected with the LAS-4000 system (GE Healthcare). Relative RNA levels were determined using Multi Gauge v3.0 (Fujifilm, Tokyo, Japan).

### Trichloroacetic acid (TCA) precipitation for protein preparation

The yeast cell pellet in a 1.5 ml tube (on ice) was resuspended with 500 μl ice-cold TCA buffer (20 mM Tris–HCl pH 8.0, 50 mM NH_4_OAc, 2 mM EDTA and 1 mM PMSF) and transferred to a new 1.5 ml tube containing 500 μl of 20% TCA and 500 μl of 0.5 mm Zirconia/Silica Beads (BioSpec Products, Bartlesville, OK, USA). The cells were vortexed for 30 s three times, and the supernatant was transferred to a new 1.5 ml tube. Another 500 μl ice-cold TCA buffer was added to a beads-containing 1.5 ml tube, vortexed for 30 s, and then the mixture of supernatant and TCA buffer was transferred to a 1.5 ml tube. After centrifugation of lysates (14 000 rpm, 15 min, 4°C), the supernatant was discarded, and the pellet was dissolved in SDS sample buffer (125 mM Tris–HCl pH 6.8, 4% SDS, 20% glycerol, 100 mM DTT, 0.01% bromophenol blue; 150 μl/6OD_600_) and heated at 95°C for 5 min (65°C for 15 min in Figure 2A). The protein solution was used for SDS-PAGE.

### Electrophoresis and western blotting

Protein samples were separated by SDS-PAGE with 12% polyacrylamide gel in 50 mM-Tris, 400 mM glycine, 0.2% SDS buffer condition, then analysed by CBB staining or transferred onto PVDF membranes (Immobilon-P, Merck Millipore, MA, USA). Membranes were blocked with 5% skim milk in PBST (10 mM Na_2_HPO_4_/NaH_2_PO_4_ pH 7.5, 0.9% NaCl and 0.1% Tween 20), incubated with primary antibodies for 1 h at room temperature, washed three times in PBST, and incubated with horseradish peroxidase (HRP)-conjugated secondary antibodies for 1 h at room temperature. For HA-tagged protein detection, the membrane was incubated with HRP-conjugated antibodies. After washing with PBST three times, chemiluminescence was detected with the LAS4000 system (GE Healthcare, Chicago, IL, USA). Primary antibodies used for western blotting were as follows: anti-HA-peroxidase was purchased from Roche (#12013819001, RRID: AB 390917); anti-FLAG M2 antibody was purchased from Sigma (# F1804-1MG, RRID: AB 262044).

### *In vitro* translation of *W(UGG)12* reporter mRNA and Hel2-mediated ubiquitination

*W(UGG)12* reporter mRNA was produced using the mMessage mMachine Kit (Thermo Fischer, Waltham, MA, USA) and used in a yeast cell-free translation extract from *uS10-3HA ski2*Δ cells. After preparation of the yeast translation extract, *in vitro* translation was performed as described previously (12). The cells were grown in YPD medium to an OD_600_ of 1.5–2.0, washed with water and 1% KCl, and finally incubated with 10 mM DTT in 100 mM Tris, pH 8.0, for 15 min at room temperature. To generate spheroplasts, 2.08 mg zymolyase per 1 g cell pellet was added to YPD medium with 1 M sorbitol and incubated for 75 min at 30°C. Spheroplasts were then washed three times with YPD/1 M sorbitol and once with 1 M sorbitol, and lysed as described previously (12) with a douncer in lysis buffer [20 mM HEPES pH 7.5, 100 mM KOAc, 2 mM Mg(OAc)_2_, 10% glycerol, 1 mM DTT, 0.5 mM PMSF and complete EDTA-free protease inhibitors (GE Healthcare)]. An S100 fraction was obtained from the lysate by low-speed centrifugation followed by ultracentrifugation of the supernatant. The S100 was passed through a PD10 column (GE Healthcare). *In vitro* translation was performed at 17°C for 60 min using an excess of template mRNA (38 μg per 415 μl extract) to prevent degradation of the resulting stalled ribosomes by endogenous response factors. For Hel2-supplemented *in vitro* translation, 32 nM Hel2 was added to the reaction. Hel2 purified from a yeast strain overexpressing *Hel2-Flag* was cultured in SC medium as described previously (12).

### Sucrose density gradient centrifugation (SDG)

Yeast cells were grown exponentially at 30°C and treated with 0.1 mg/ml cycloheximide for 5 min before harvesting. Cells were harvested by centrifugation, and the cell pellet was frozen and ground in liquid nitrogen using a mortar. The cell powder was resuspended with lysis buffer [20 mM HEPES–KOH pH 7.4, 100 mM potassium acetate, 2 mM magnesium acetate, 0.5 mM DTT, 1 mM phenylmethylsulfonyl fluoride and 1 tablet/10 ml complete mini EDTA-free (#11836170001, Roche)] to prepare the crude extracts. Sucrose gradients (10–50% sucrose in 10 mM Tris-acetate pH 7.4, 70 mM ammonium acetate and 4 mM magnesium acetate) were prepared in 25  ×  89 mm polyallomer tubes (Beckman Coulter) using a Gradient Master (BioComp). Crude extracts (the equivalent of 50 *A*_260_ units) were layered on top of the sucrose gradients and centrifuged at 150 000 × g in a P28S rotor (Hitachi Koki, Japan) for 2.5 h at 4°C. The gradients were then fractionated with BioComp Piston Gradient Fractionator. The polysome profiles were generated by continuous absorbance measurement at 254 nm using a single path UV-1 optical unit (ATTO Biomini UV-monitor) connected to a chart recorder (ATTO digital mini-recorder). For the western blots shown in Figures 2F and 4C, 900 μl of each fraction was mixed with 180 μl of 100% TCA and incubated for 15 min at 4°C. After centrifugation (14 000 rpm, 15 min, 4°C), the supernatant was removed, and the pellet was dissolved in SDS sample buffer (125 mM Tris–HCl pH 6.8, 4% SDS, 20% glycerol, 100 mM DTT, 0.01% bromophenol blue, 150 μl/6OD_600_) and heated at 65°C for 15 min. The resulting solution was separated by SDS-PAGE.

### Quantification and statistical analysis

The levels of RNA were quantified using Multi Gauge v3.0 (Fujifilm). All blot experiments were repeated three times independently, and a representative result is shown. Quantitative data are presented as the mean ± standard deviation (SD) from at least three independent experiments.

## RESULTS

### Consecutive tryptophan codons induce RQC2-independent RQC

**Figure 1.**
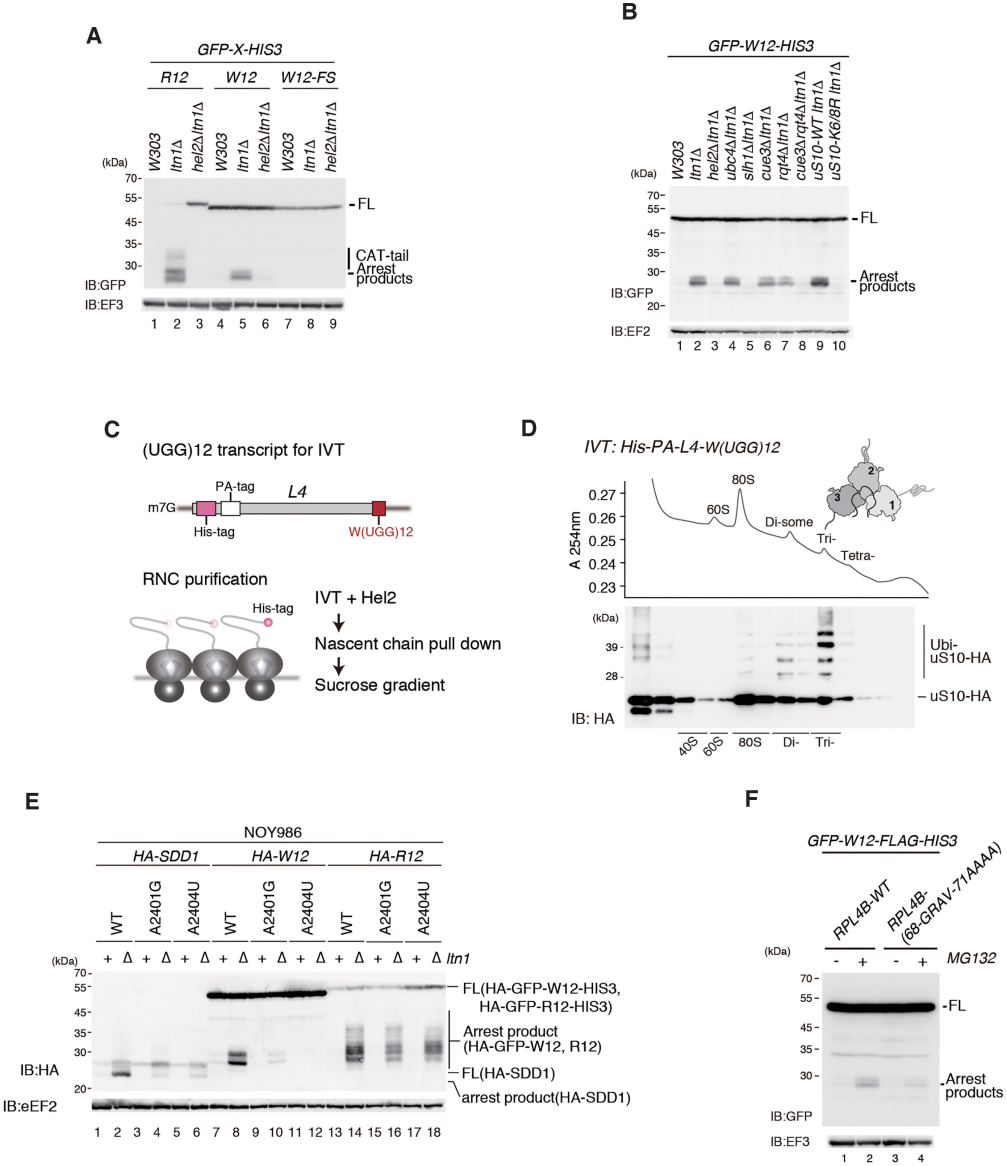
Consecutive tryptophan codons induce Rqc2-independent RQC. (**A**) Consecutive basic amino acids or tryptophan sequences induce ribosome-associated quality control (RQC). Cells harbouring the indicated p*GPDp-GFP-X-HIS3* plasmid were grown in SC-Ura medium, and the samples were analysed by western blotting with anti-GFP or anti-EF3 antibody. (**B**) Rqt1/Hel2-dependent ubiquitination of uS10 and of the RQC-trigger (RQT) complex is required for RQC induced by consecutive tryptophan codons. The protein samples were prepared from the indicated mutant cells containing the *GFP-W(UGG)12-HIS3* reporter and were subjected to western blot analysis. (**C**) Schematic drawing of the *L4-W(UGG)12* transcript used in the *in vitro* translation (IVT) assay (upper panel). Scheme of ribosome-nascent polypeptide complex (RNC) purification (lower panel). (**D**) RNCs with *L4-W(UGG)12* were prepared by IVT with addition of purified Hel2, separated by sucrose density gradient and detected by UV absorbance at 254 nm wavelength. Tagged uS10 in each fraction was detected by immunoblotting using anti-HA antibody. (**E**) Mutational analysis of 25S rRNA. The indicated 25S rRNA mutant variants were expressed from a plasmid in an *rDNA* deletion strain (NOY986)(12). Protein samples were prepared from the indicated cells containing *HA-SDD1*, *HA-GFP-W12-HIS3* and *HA-GFP-R12-HIS3* plasmids, and were analysed by western blotting. (**F**) The levels of the arrest products derived from *GFP-W(UGG)12-HIS3* were decreased in the uL4 mutant cells. The indicated *uL4* alleles were expressed from plasmids in the *uL4*Δ (*uL4AΔuL4BΔ*) strain (12).

We previously demonstrated that consecutive tryptophan codons induce translation arrest leading to proteasomal degradation (32). The E3 ubiquitin ligase Ltn1 catalyses the ubiquitination of the arrested products derived from the *R(CGN)12* reporter on the 60S subunit, thereby targeting them for degradation by the proteasome. In this study, we detected arrest products derived from the *GFP-W(UGG)12-HIS3* reporter compared with the *GFP-R(CGN)12-HIS3* reporter (Figure 1A). Arrest products derived from the *W(UGG)12* reporter were detected in *ltn1*Δ cells, indicating that the 12 consecutive tryptophan codons induce RQC (Figure 1A). The *GFP-W12-FS-HIS3* reporter is identical to the *GFP-W12-HIS3* reporter except for an additional nucleotide just before the sequence encoding polytryptophan and two additional nucleotides following the sequence. This frameshift mutation eliminated the arrest products, indicating that the consecutive tryptophan amino acid sequence, and not the nucleotide sequence, is necessary for inducing RQC. It could not be excluded that a frameshift abolishes the pairing of mRNA and tRNA, as well as change the amino acid sequence, and may lead to the effect.

The E3 ubiquitin ligase Hel2 is involved in translation arrest induced by polyarginine sequences. The W(UGG)12 reporter assay showed that arrest products were eliminated in the *hel2*Δ*ltn1*Δ double mutants, indicating that RQC induced by polytryptophan requires the E3 ligase as well as R(CGN)12 (Figure 1A, lanes 3, 6 and 9). We previously reported that the RQT complex, composed of the RNA helicase-family protein Slh1, the ubiquitin-binding protein Cue3 and Rqt4, is required for RQC induced by the R(CGN)12 reporter (5,12). To determine the involvement of the RQT complex in RQC induced by W(UGG)12 reporters, we measured the levels of arrest products in each deletion mutant (Figure 1B). Although arrest products were detected in single deletion mutants of *cue3*Δ and *rqt4*Δ, arrest products were not detected in the *cue3*Δ*rqt4*Δ double mutant, indicating that the RQT complex is essential for RQC induced by consecutive tryptophan codons such as the R(CGN)12 sequence (Figure 1B, lanes 6–8). RQT is reported to be composed of three subunits, Slh1, Cue3 and Rtq4 in yeast (5,12), and the conserved function of mammalian homologous of the yeast subunits was demonstrated (13,14). RQC induced by R12 sequence also completely depends on Slh1. The arrest products were partially reduced in *cue3*Δ and *rqt4*Δ single mutants, but almost diminished in *cue3*Δ*rqt4*Δ double mutant cells, indicating that Cue3 and Rqt4 may independently and redundantly contribute to RQC. In this study, RQC induced by W12 demonstrated the same dependency to the subunits of the RQT complex, suggesting that RQT-dependent subunit dissociation induced by R12 and W12 may be the same mechanism. To examine the ubiquitination of uS10 on the RQC pathway, a reporter assay was performed in the *uS10-K6/8R* mutant defective in uS10 ubiquitination. The results showed that arrest products were abolished in the *uS10-K6/8R* mutant, as observed in the *hel2*Δ mutant. This suggests that Hel2-dependent ubiquitination of uS10 at K6 and K8 is essential for the activation of RQC systems (Figure 1B, lanes 9 and 10).

Next, we performed ribosome stalling-coupled ubiquitination using an *in vitro* translation reaction as previously reported (10,12). To purify the colliding RNCs, we used a cell-free *in vitro* translation system with *PA-His-L4-W(UGG)12* model mRNA, which contains consecutive tryptophan codons and a sequence coding for two N-terminal epitope tags: a His-tag and a PA-tag (Figure 1C). Ribosomes translating the *W(UGG)12* reporter mRNA stalled and formed tri-ribosomes, and Hel2 preferentially ubiquitinated trisomes (Figure 1D). This result is consistent with previous results from our group obtained with an endogenous stalling sequence of *SDD1* mRNA (12). The specific interaction between the nascent peptide and the stalled ribosome in the exit tunnel is also crucial to induce RQC by *SDD1* (12). Juxtaposed on the tunnel side, the nucleotides A2401 (A2059 in *Escherichia coli*) and A2404 (A2062 in *E. coli*) were exposed near the nascent polypeptide (NC), and mutation of these bases to G and U, respectively, impaired RQC induction by the W12 stalling sequence (Figure 1E) as observed for *SDD1* stalling (12). Mutation of the uL4 loop (^68^GRAV^71^ to AAAA) suppressed translational arrest and RQC by the W12 sequence (Figure 1F). This indicates that the interaction between consecutive tryptophan codons and ribosome tunnel was crucial for stalling and RQC by W12 similar to *SDD1* stalling (12).

### The consecutive tryptophan codons inhibit CAT-tailing by CGA codon cluster

**Figure 2.**
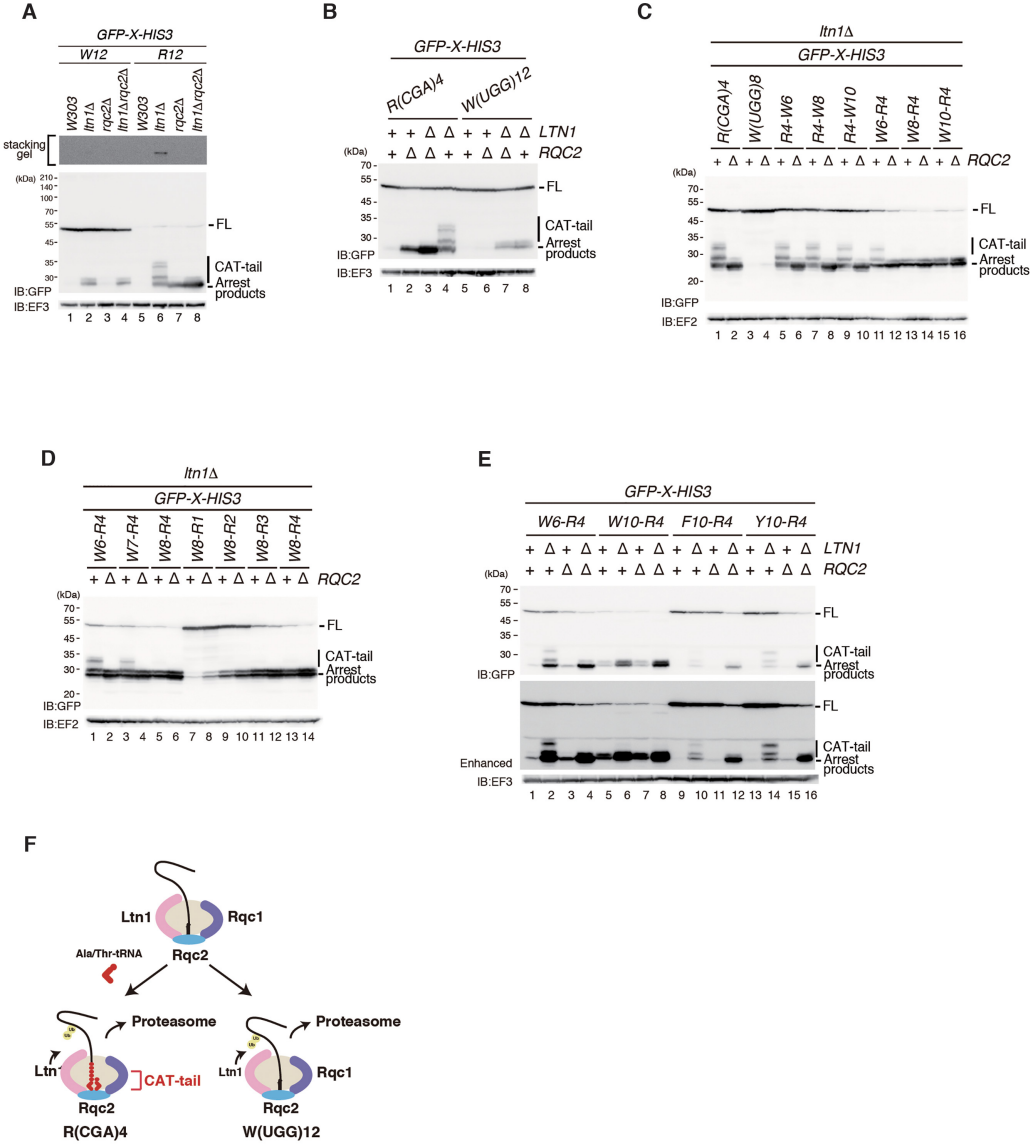
Consecutive tryptophan codons inhibit CAT-tailing by CGA codon cluster. (**A**) High molecular weight proteins expressed in *ltn1Δ* mutant cells are not detected in the W(UGG)12 reporter. (**B**) Consecutive tryptophan codons induce RQC without CAT-tailing. Cells harbouring *pGPDp-GFP-R(CGA)4-HIS3* or *pGPDp-GFP-W(UGG)12-HIS3* plasmids were grown in SC-Ura medium, and the samples were analysed by western blotting with anti-GFP or anti-EF3 antibody. (**C**) More than eight consecutive tryptophan residues located at the PTC inhibit CAT-tailing. (**D**) Consecutive tryptophan codons located near the PTC inhibit CGA codon-mediated CAT-tailing. (**E**) Consecutive tryptophan, but not phenylalanine or tyrosine sequences, inhibit CAT-tailing by R(CGA)4. (**F**) Model of the inhibition of CAT-tailing by consecutive tryptophan residues.

Contrary to RQC induced by tandem CGA codons (R12), translation arrest induced by polytryptophan sequences produces arrest products without CAT-tailing in *ltn1*Δ cells (Figure 1A, lanes 2 and 5). Rqc2 is required for efficient recruitment of Ltn1 to the 60S subunit (18–20, 27,28). Moreover, Rqc2 engages the stalled nascent chain and recruits tRNAs charged with alanine and threonine, suggesting that the 60S subunit catalyses CAT-tail formation in an unusual reaction. Poly-basic amino acids (R12) or tandem CGA codons such as R(CGA)4 induce translation arrest, and Rqc2-dependent CAT-tailing is observed in *ltn1*Δ cells (Figure 2A, lane 6; Figure 2B, lane 4). However, compared with RQC induced by R12 or R(CGA)4, translation arrest induced by polytryptophan sequences produces arrest products without CAT-tailing in *ltn1*Δ cells (Figure 2A, lane 2; Figure 2B, lane 8). In addition, arrest products are not detected in *rqc2*Δ single mutant cells in the W(UGG)12 reporter (Figure 2A, lane 3; Figure 2B, lane 6). Therefore, we propose that polytryptophan sequences induce Rqc2-independent RQC without CAT-tailing. A previous study demonstrated that the CAT-tail mediates the formation of SDS-resistant aggregates along with the polylysine tract present in nonstop proteins. In the R(CGN)12 reporter system, we confirmed the aggregation in *ltn1*Δ cells (Figure 2A, lane 6). However, in the W(UGG)12 reporter, this high molecular weight protein was not detected, indicating that the arrest products without the CAT-tail may not form aggregates (Figure 2A, lane 2).

We hypothesised that consecutive tryptophan residues located at the peptidyl transferase center (PTC) or the discrimination gate of the ribosomal tunnel inhibit CAT-tailing. More than 11 consecutive tryptophan codons induced RQC without CAT-tailing (Figure 2B, lanes 7 and 8; Supplementary Figure S1). By contrast, the CGA codon cluster (CGA)4 efficiently induced RQC with CAT-tailing (Figure 2B, lane 4). We examined whether consecutive tryptophan residues inhibited CAT-tailing when translation arrest was induced by R(CGA)4 (Figure 2C). In *GFP-R4-W6-HIS3*, *GFP-R4-W8-HIS3* and *GFP-R4-W10-HIS3* reporters, polytryptophan sequences are not translated because translation arrest is induced by R4, and we observed the addition of CAT-tails to the arrest products (Figure 2C, lanes 5–10). However, *GFP-W8-R4-HIS3* and *GFP-W10-R4-HIS3* reporters produced arrest products without CAT-tailing (Figure 2C, lanes 13–16). In the *GFP-W6-R4-HIS3* reporter, CAT-tailing was detected, suggesting that more than eight consecutive tryptophan residues located near the PTC inhibited CAT-tailing. We also examined the effects of the cluster of tryptophan residues before the CGA codons on RQC and CAT-tailing (Figure 2D). In the *GFP-W7-R4-HIS3* reporter, CAT-tailing was detected, indicating that more than eight consecutive tryptophan residues located near the PTC inhibited CAT-tailing (Figure 2D, lanes 1–6; Figure 2E). We inserted CGA codons downstream of the eight consecutive tryptophan residues, and even a single CGA codon weakly induced RQC (Figure 2D, lanes 7–14). This is consistent with a composite stalling mechanism as demonstrated by AAA cluster and *SDD1* stalling, in which the NC-induced interference with the PTC is combined with a difficult-to-decode mRNA for cooperative stalling (12). In the *GFP-W8-R4-HIS3* construct, efficient ribosome stalling results in RQC2-independent of RQC without CAT-tailing (Figure 2D, lanes 13 and 14). The levels of arrest products with or without CAT-tailing derived from the indicated reporters in W303, *ltn1*Δ, *rqc2*Δ, *ltn1*Δ*rqc2*Δ mutant cells were determined by western blot analysis (Figure 2E). The arrest products derived from these reporters were stabilized in *ltn1*Δ background. In RQC induced by W6-R4, polyphenylalanine (F10)-R4 and polytyrosine (Y10)-R4, the CAT-tailed arrest products were detected (Figure 2E). In contrast, the CAT-tailed arrest products were not detected in RQC by W10-R4, suggesting that F10 and Y10 sequences did not inhibit CAT-tailing induced by the R(CGA)4 sequence, suggesting that polyphenylalanine (F10) and polytyrosine (Y10) sequences did not inhibit CAT-tailing induced by the R(CGA)4 sequence (Figure 2E). We propose that polytryptophan sequences have a specific inhibitory effect on Rqc2-dependent CAT-tailing (Figure 2F).

### Inhibition of CAT-tailing depends on the position of polytryptophan in peptidyl-tRNA

**Figure 3.**
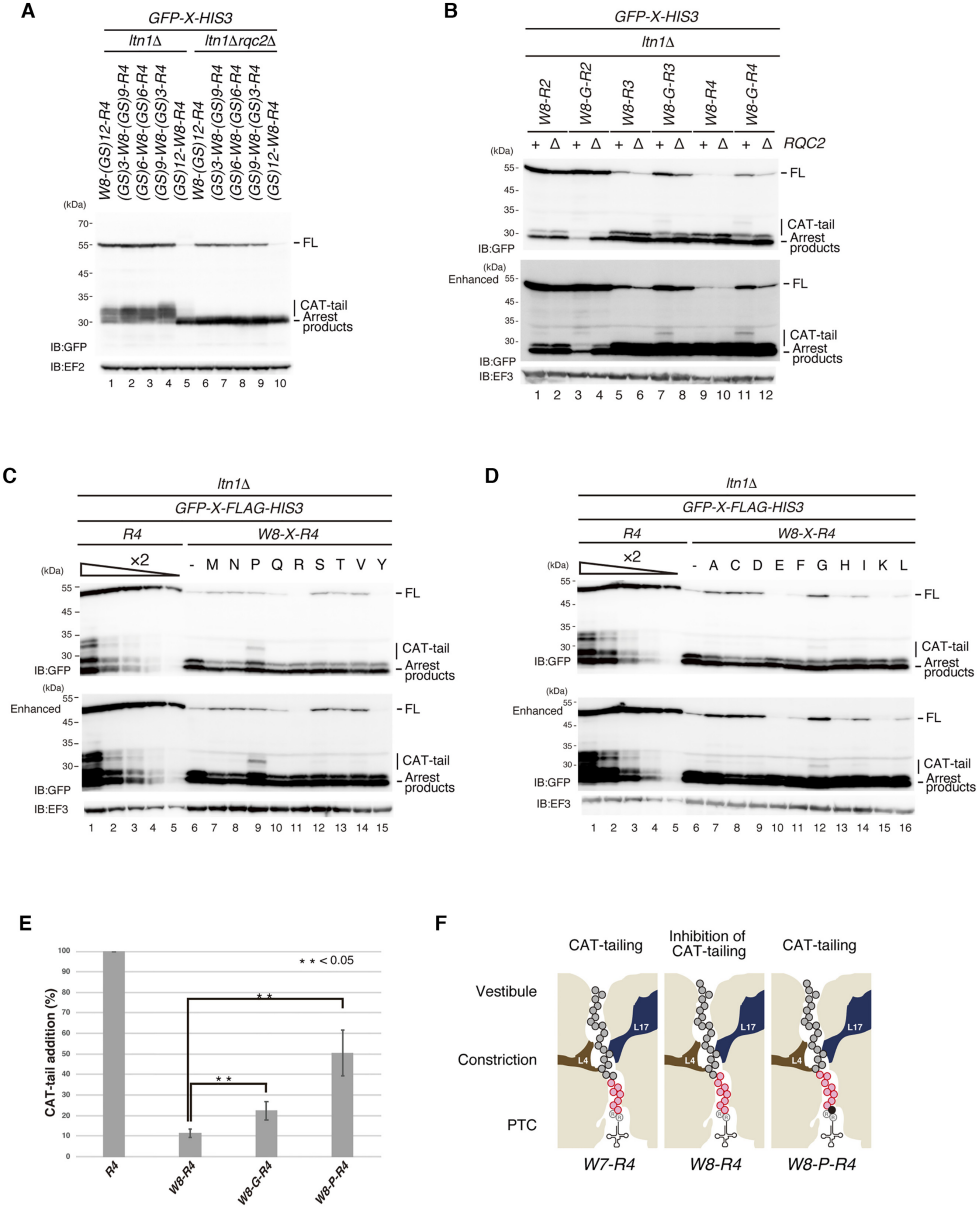
Proper positioning of more than eight consecutive tryptophan codons on peptidyl-tRNA in the 60S subunit is required to inhibit CAT-tailing. (**A**) The insertion of GS residues between W(UGG)8 and R(CGA)4 abolished the inhibitory function of W(UGG)8 on CAT-tailing. The protein samples were prepared from W303*ltn1*Δ or W303*ltn1*Δ*rqc2*Δ cells containing the indicated reporters. Western blot analysis was performed to evaluate the effect of glycine residue insertion on decreasing the inhibitory effect of W(UGG)8 on CAT-tailing. (**B**) The glycine residue between W(UGG)8 and R(CGA)2 or R(CGA)3 abolished the inhibitory effect of W(UGG)8 on CAT-tailing. The protein samples were prepared from W303*ltn1*Δ or W303*ltn1*Δ*rqc2*Δ cells containing the indicated reporters. Western blot analysis was performed to evaluate the effect of glycine residue insertion on decreasing the inhibitory effect of W(UGG)8 on CAT-tailing. (**C**, **D**) Glycine and proline residue(s) between W(UGG)8 and R(CGA)4 abolished the inhibitory function of W(UGG)8 on CAT-tailing. The protein samples were prepared from W303*ltn1*Δ cells containing the *GFP-W8-X-R4-HIS3* reporter. Western blot analysis was performed to evaluate the effect of the insertion of single residues on decreasing the inhibitory effect of W(UGG)8 on CAT-tailing. (**E**) Quantification of the efficiency of CAT-tailing in the reports shown in (C) and (D). The CAT-tailing efficiency was calculated as the ratio of [arrest products] + [CAT-tailed products] to [CAT-tailed products]. Three replicates were used to calculate the mean and SD. (**F**) Schematic drawing of nascent peptidyl-tRNA containing polytryptophan and arginine residues in the ribosome tunnel. The red circles indicate tryptophan residues, and open circles with R indicate arginine residues; the residue marked in black is Proline.

The above results demonstrate that more than eight consecutive tryptophan residues located near the PTC inhibit CAT-tailing. Next, we confirmed whether CAT-tailing inhibition is affected by the location of consecutive tryptophan residues. We constructed reporters by inserting consecutive glycine-serine (GS) sequences upstream or downstream of W(UGG)8, and 12 GS sequences were inserted upstream of R(CGA)4 to induce RQC (Figure 3). W(UGG)8 just upstream of R(CGA)4 efficiently inhibited CAT-tailing (Figure 3A, lanes 5 and 10), whereas the insertion of (GS)12, (GS)9, (GS)6, and (GS)3 between W(UGG)8 and R(CGA)4 abolished the inhibitory function of W(UGG)8 on CAT-tailing (Figure 3A, lanes 1–4 and 6–9). These results suggest that the position of W(UGG)8 relative to the PTC is crucial for inhibiting CAT-tailing. We then constructed *GFP-W8-G-R(2–4)-HIS3* reporters containing a single glycine residue (G) between the CAT-tail-inhibitory element (W8) and the CAT-tail-inducing CGA codons [R(2–3)]. Two or three CGA codons downstream of eight consecutive tryptophan residues efficiently induced RQC (Figure 3B, lanes 1–2 and 5–6), and the insertion of a single glycine residue abolished the inhibitory effect of W(UGG)8 on CAT-tailing (Figure 3B, lanes 3–4, 7–8 and 11–12). We then aimed to identify the residue(s) between W(UGG)8 and R(CGA)4 critical for abolishing the inhibitory function of W(UGG)8 on CAT-tailing. To this end, we constructed the *GFP-W8-X-R4-HIS3* reporter. Insertion of a single proline residue greatly diminished the inhibitory effect of W(UGG)8 on CAT-tailing (Figure 3C, lane 9; quantification in Figure 3E), and insertion of a single glycine residue moderately but significantly reduced the inhibitory effect of W(UGG)8 on CAT-tailing (Figure 3D, lane 12; Figure 3E). These results suggest that the inhibitory effect of W(UGG)8 on CAT-tailing depends on the position of polytryptophan in the peptidyl-tRNA at the P-site. Mass spectrometry of the arrest products derived from the R(CGN)12 reporter in *ltn1*Δ mutant cells revealed that RQC takes place when the ribosome translates the second and third CGA codons (5). Ribosome profiling also detected the accumulation of ribosome footprints in the second and third CGA codons (5). This suggests that translation of W(UGG)8-R(CGA)4 reporter mRNAs causes ribosome stalling at the second and third CGA codons, and peptidyl-tRNA containing consecutive arginine residues and eight tryptophan residues inhibits CAT-tailing in the 60S subunit (Figure 3F, left panel). Neither seven tryptophan residues nor the insertion of proline residues downstream of W(UGG)8 inhibited CAT-tailing in the 60S subunit, suggesting that the proper positioning of the eight tryptophan residues in the ribosome tunnel is crucial to inhibit CAT-tailing (Figure 3F, middle and right panels).

### Consecutive tryptophan codons induce endonucleolytic cleavage (NGD)

**Figure 4.**
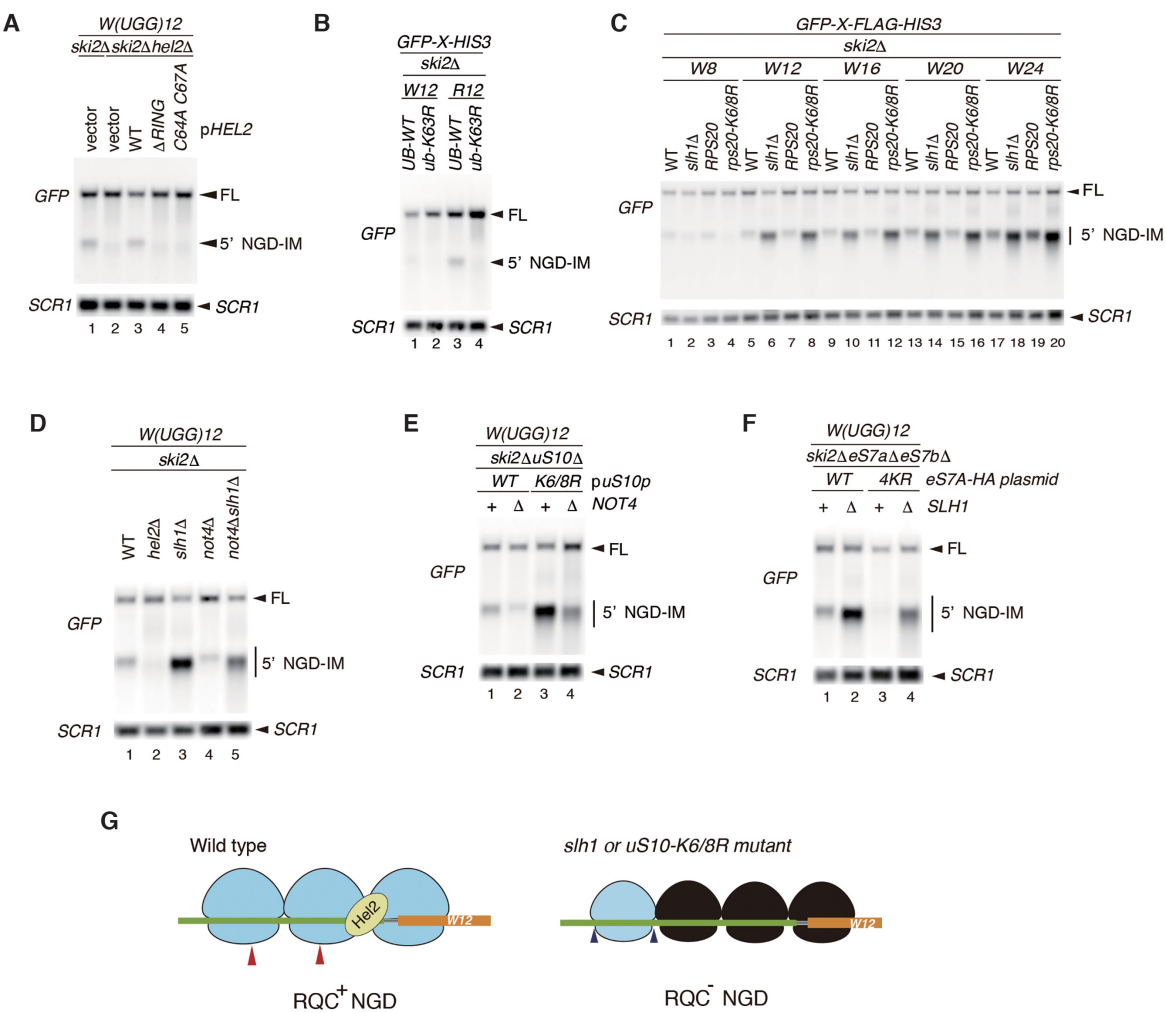
Consecutive tryptophan codons induce no-go decay (NGD). (**A**, **B**) Consecutive tryptophan codons induce NGD in the Hel2-mediated uS10 ubiquitination-dependent manner. RNA samples were prepared from cells and analysed by Northern blotting with DIG-labelled *GFP* or *SCR1* probes. (**C**) More than eight consecutive tryptophan codons induced NGD. (**D**) 5′-NGD-IMs derived from GFP-W(UGG)12-HIS3 were detected in the indicated mutant cells by northern blotting with a DIG-labeled GFP probe. (**E**) Not4 increased the level of 5′-NGD-IMs derived from *GFP-W(UGG)12-HIS3* in *uS10-K6/8R* mutant cells. (**F**) Not4-mediated eS7 ubiquitination increased the level of 5′-NGD-IMs derived from *GFP-W(UGG)12-HIS3* in *slh1* mutant cells. (**G**) The proposed model for endoribonucleolytic cleavages induced by consecutive tryptophan codons-mediated ribosome stalling. The red arrowheads in the left panel indicate the putative endoribonucleolytic cleavage sites in wild type. The blue arrowheads in the right panel indicate the putative cleavage sites in the mutants defective in the uS10 ubiquitination (uS10-K6/8R) or Slh1, an essential factor for the RQT-mediated subunit dissociation (slh1). Based on the reconstitution with collided ribosomes on *SDD1* mRNA, it has been demonstrated that the leading and stalling ribosome is ubiquitinated and dissociated into subunits by the RQT complex (12). eS7 ubiquitination by Not4 and Hel2 E3 ligases are required for NGD^RQC-^ (10). Although it is strongly suggested that the leading ribosome in the trisome formed by *SDD1-*stalling sequence is ubiquitinated and dissociated into the subunits by the RQT complex (12), it is still unknown which collided ribosomes are ubiquitinated in the UGG codons-inducing substrates. There the position and species of the ubiquitinated ribosomes are not demonstrated in the model.

The results of this study suggest that polytryptophan sequences induce translation arrest leading to proteasomal degradation. We therefore examined whether polytryptophan sequences also induce NGD, the mRNA surveillance pathway. To address this possibility, we detected intermediate endonucleolytic mRNA cleavage products, 5′ No-Go-Decay intermediate (5′-NGD-IM). The 5′-NGD-IM derived from arrest sequences is normally degraded by the exosome and can be detected in mutants lacking the exosome co-factor Ski2 (10,29). In *ski2*Δ mutant cells, 5′-NGD-IM derived from W(UGG)12 was detected in *ski2*Δ mutant cells (Figure 4A, lane 1), suggesting that consecutive tryptophan codons induced NGD. To determine the minimum number of tryptophan residues necessary for induction of NGD, we changed the number of consecutive tryptophan residues inserted between *GFP* and *HIS3*. The results showed that more than eight consecutive tryptophan codons induced NGD (Supplementary Figure S2B). The 3′-NGD-IM, which is normally degraded by the 5′-3′ exonuclease Xrn1, was detected in *xrn1*Δ mutant cells (Supplementary Figure S2C). In a previous study from our group, we reported that Hel2 is not only required for degradation of the aberrant peptide by the RQC system, but also for the decay of aberrant mRNA by the NGD pathway. Hel2-mediated ubiquitination of the ribosome is required both for canonical NGD (NGD^RQC+^) and RQC coupled to the disome, and RQC-uncoupled NGD outside the disome (NGD^RQC−^) can occur in a Not4-dependent manner (10). Consistently, 5′-NGD-IM was not detected in *ski2*Δ*hel2*Δ mutant cells (Figure 4A). To obtain a more detailed insight into the sections of Hel2 involved in NGD and RQC, deletions were introduced into the Hel2 sequence. The 5′-NGD-IM and the peptide arrest products did not accumulate in the *hel2* mutants carrying deletions of the RING domain (ΔRING) or alanine substitution of the conserved zinc-finger (ZnF) cysteine residues (C64A C67A) within the RING domain, indicating loss of function (Figure 4A, lanes 4 and 5). To determine whether K63-linked ubiquitin chains are critical for NGD, we used the *ub-K63R* mutant cell strain in which all endogenous ubiquitin-encoding genes are modified and only the K63R mutant ubiquitin was expressed (10). The level of 5′-NGD-IM derived from the *R(CGN)_12_* reporter mRNA was measured in an isogenic *UB-WT* strain with *ski2* deletion and in ubiquitin mutant *ski2*Δ*ub-K63R* cells. The level of 5′-NGD-IM was significantly decreased in *ski2*Δ*ub-K63R* mutant cells (Figure 4B), indicating that endonucleolytic cleavage of mRNA in NGD required K63-linked ubiquitination.

We recently reported that *slh1*Δ or *uS10-K6/8R* mutant cells produce other endonucleolytic mRNA cleavage sites, which are located >45–51 nucleotides upstream of the normal cleavage site, as determined using the R(CGN)12 reporter assay (10). In the W(UGG)12 reporter assay, the cleavage site was shifted upstream in *slh1*Δ or *uS10-K6/8R* mutant cells (Figure 4C, lanes 6, 8, 10, 12, 14, 16, 18 and 20; Supplementary Figure S2C). This shift was also observed in *xrn1*Δ mutant cells (Supplementary Figure S2D). Therefore, consecutive tryptophan sequences induce NGD via the same pathway as R(CGN)12, with RQC-coupled NGD (NGDRQC+) and RQC-uncoupled NGD (NGDRQC–) happening.

Not4-dependent ubiquitination is required for NGD^RQC−^ outside the disome (10). We therefore examined whether Not4-dependent ubiquitination of eS7A is required for NGD^RQC−^ by W12 in RQC-defective *slh1/rqt2*Δ or *uS10-K6/8R* mutant cells. The level of the shorter 5′-NGD-IM derived from the W(UGG)_12_ reporter was slightly lower in *ski2*Δ*slh1*Δ*not4*Δ cells than in *ski2*Δ*slh1*Δ cells (Figure 4D, lanes 3 and 5), and the shorter 5′-NGD-IMs were also reduced in *ski2*Δ*not4*Δ*uS10-K6/8R* cells compared with the levels in *ski2*Δ*uS10-K6/8R* cells (Figure 4E; lanes 3 and 4, respectively). These results indicate that Not4 plays an important role in NGD^RQC−^ induced by W(UGG)_12_. We then determined the levels and size of 5′-NGD-IMs derived from the *W(UGG)_12_* reporter in *ski2*Δe*S7A*Δ*eS7B*Δ or *ski2*Δ*slh1*Δ*eS7A*Δ*eS7B*Δ mutant cells harbouring plasmids expressing either eS7A wild-type (WT) or eS7A-4KR mutant (4KR). The shorter 5′-NGD-IM in *slh1*Δ*eS7A-WT* was produced by NGD^RQC−^, and its level was higher than that produced by NGD^RQC+^ in eS7A-WT cells (Figure 4F, lanes 1 and 2). The level of the shorter 5′-NGD-IM was lower in the *slh1*Δ*eS7A-4KR* mutant than in *slh1*Δ*eS7A-WT* (Figure 4F, lanes 2 and 4), confirming that Not4-mediated ubiquitination of eS7A plays a crucial role in endonucleolytic cleavage. Taken together, these results suggest that similar to NGD by *R(CGN)_12_*, endonucleolytic cleavage by *W(UGG)_12_* in NGD^RQC−^ outside the disome requires stepwise ubiquitination of eS7A by the E3 ligases Not4 and Hel2 (Figure 4G).

## DISCUSSION

Ribosome stalling during translation elongation induces quality control systems to prevent the production of harmful products. We previously reported that consecutive tryptophan codons induce translation arrest leading to proteasomal degradation (32); however, the underlying mechanisms and factors involved remain unknown.

First, we detected arrest products derived from polytryptophan sequences in *ltn1*Δ mutant cells. Detectable levels of arrest products were only present in the *ltn1*Δ mutant, although the levels were not as high as those produced by R(CGN)12 (Figure 1). After introduction of a frameshift mutation, the arrest products were undetectable (Figure 1A), suggesting that translation of consecutive tryptophan residues results in translation arrest, leading to nascent polypeptide degradation. We showed that the steric hindrance between polytryptophan residues and the discrimination gate of the ribosomal tunnel induced RQC (Figure 1D and E). Mutation of a residues at 68-GRAV-71 of uL4/RPL4, which is located at the discrimination gate and is assumed to expand the narrow discrimination gate, decreased the levels of arrest products derived from W(UGG)_12_-mediated translation arrest (Figure 1E), suggesting that the translation arrest was resolved. These results suggest that consecutive tryptophan codons induce ribosome stalling via interaction with the ribosome tunnel and RQC.

We demonstrated that eight consecutive tryptophan residues located near the PTC inhibit CAT-tailing. The presence of a few CGA codons downstream of the polytryptophan sequence did not lead to CAT-tailing (Figures 1 and 2). One possibility is that arginine codons are not actually translated at all in the construct. However, the introduction of even a single glycine or proline codon between the two clusters allows for CAT-tailing to resume (Figure 3B–D), indicating that the W8 element inhibits the CAT-tailing but not translation of four CGA codons itself.

The insertion of a single glycine or proline residues between W8 and R4 affected CAT-tailing, but most other amino acid bases did not. The specificity of CAT-tailing inhibition may be derived from steric hindrance between nascent polypeptides and the ribosomal tunnel. After subunit dissociation, nascent polypeptides pass through the ribosomal tunnel to elongate the CAT-tail. However, polytryptophan residues in the discrimination gate of the ribosomal tunnel may prevent nascent polypeptides from passing through the tunnel. Moreover, arrest products derived from W(UGG)12 were not detected in the *rqc2*Δ mutant (Figure 2B), suggesting that Rqc2 is not required for RQC by consecutive tryptophan codons. Although Rqc2 is required for efficient recruitment of Ltn1, Ltn1 ubiquitinates polytryptophan peptides efficiently in the absence of Rqc2. One possible explanation is that Ltn1 binds to the polytryptophan peptide–60S complex because of its stable structure due to the strong interaction between polytryptophan peptides and the ribosome tunnel. The insertion of polytryptophan peptides into the ribosomal tunnel may hinder Rqc2-mediated formation of the CAT-tail. The polyphenylalanine (F10) or polytyrosine (Y10) sequences still requires Rqc2 for RQC (Figure 2E), suggesting that the interaction between these peptides and the ribosome tunnel may not be stable enough for the binding of Ltn1 to 60S subunit without Rqc2 and for the inhibition of CAT-tail formation by Rqc2. The possibility that the polytryptophan peptide-60S complex inhibits the binding of Rqc2 to the 60S complex cannot be excluded.

Our results suggest that the inhibitory effect of the polytryptophan peptides on the Rqc2-dependent CAT-tail formation requires precise positioning in the ribosome tunnel. The insertion of a single glycine or proline residue between polytryptophan peptide and stalling inducing tandem CGA codons suppresses the inhibitory effect of polytryptophan peptides (Figure 3). One possibility is that the precise positioning of the tryptophan residue in the vicinity of the peptidyl-transferase center is crucial to prevent the peptide-bond formation to elongate CAT-tail.

Hel2-mediated polyubiquitination of 40S subunit ribosomal proteins is crucial to trigger both endonucleolytic cleavage and RQC, thus acting as a master regulator of both pathways (10,30,31). It has been proposed that RQC dissects the NGD pathway into two branches depending on the targets of Hel2-mediated polyubiquitination (10). The first branch is coupled to RQC (NGD^RQC+^) and depends on uS10 ubiquitination, leading to cleavage events in the mRNA covered by the first two stalled ribosomes (disome unit) (10). The second branch is uncoupled from RQC (NGD^RQC−^) and depends on eS7 ubiquitination followed by Not4-mediated monoubiqutination, resulting in upstream cleavage events on the mRNA outside the disome unit and potentially covered by following ribosomes. NGD could be as efficient in the alternative NGD^RQC-^ pathway as in the ‘canonical’ NGD^RQC+^ pathway, which strongly argues in favour of its physiological relevance. The present results demonstrate that W(UGG)_12_-mediated translation arrest induces Rqc2-independent RQC (Figures 1–3) and both branches of NGD (Figure 4). We therefore propose that translation arrest by the nascent peptide results in the formation of the trisome to trigger RQC steps by the recruitment of Hel2 to induce both polyubiquitination of 40S subunit ribosomal proteins on the collided ribosomes and endonucleolytic cleavage at the vicinity of collided ribosomes. This study reveals the novel function of nascent peptides in the 60S subunit that specifically modulates the Rqc2-dependency of the reactions in the 60S subunit, CAT-tail formation and Ltn1-mediated polyubiquitination.

## QUANTIFICATION AND STATISTICAL ANALYSIS

The levels of 18S rRNA were quantified using Multi Gauge (v3.0, Fujifilm). Quantified and calculated half-lives represent mean ± SD from three independent experiments. All blot experiments were repeated at least twice, and a representative result is shown.

## Supplementary Material

gkab005_Supplemental_Files
