# Supplementary material for: The nascent polypeptide in the 60S subunit determines the Rqc2-dependency of ribosomal quality control
Source: Nucleic Acids Res. 2021 Jan 28;49(4):2102–13. doi: 10.1093/nar/gkab005 (PMC7913769; doi:10.1093/nar/gkab005)
Supplement: gkab005_Supplemental_Files [file gkab005_supplemental_files.zip › NAR-02902-V-2020.R1 Supplementary_Figures.pdf]

Supplementary Materials for

The nascent polypeptide in the 60S subunit determines the Rqc2-dependency of ribosomal quality control

Masato Mizuno[1,#], Shuhei Ebine[1,#], Okuto Shounai[1], Shizuka Nakajima[1], Shota Tomomatsu[1], Ken Ikeuchi[1], Yoshitaka Matsuo[1], and Toshifumi Inada[1*]

[1]Graduate School of Pharmaceutical Science, Tohoku University, Aoba-ku, Sendai 980-8578, Japan.

[#]These authors contribute to this study equally.

To whom correspondence should be addressed: Toshifumi Inada, Sendai 980-8578, Japan. Tel: +81 (22) 795-6874 Fax: +81 (22) 795-6873 E-mail:toshifumi.inada.a3@tohoku.ac.jp

This PDF file includes:

Supplementary Figures 1 to 2

Supplementary Tables 1 to 2

Mizuno et al Supplementary Figure 1

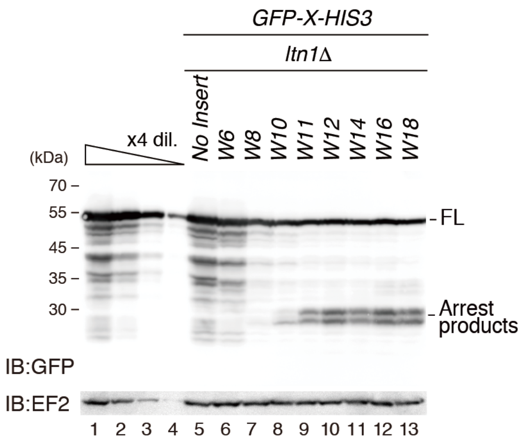

Supplementary Figure 1. More than eleven consecutive tryptophan sequences induce RQC without CAT-tailing. The proteins samples were prepared from W303*ltn1*Δ cells containing the *GFP-X-HIS3* reporters. Western blot analysis was performed to evaluate RQC induced by Consecutive tryptophan sequences.

Mizuno et al Supplementary Figure 2

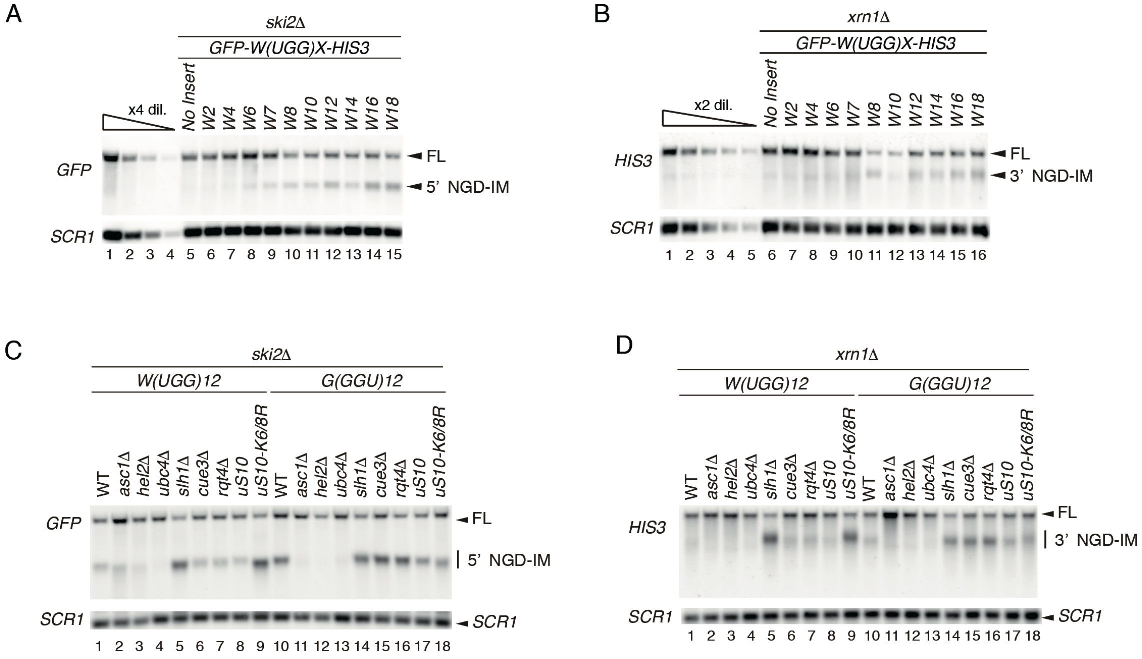

Supplementary Figure 2. **More than eight consecutive tryptophan sequences induce NGD**. **(A)** The 5' NGD-IM, which is normally degraded by the Ski complex composed of Ski2/Ski3. Ski8 and 3'-5' exonuclease complex, are detected in *ski2*Δ mutant cells. **(B)** The 3' NGD-IM, which is normally degraded by the 5'-3' exonuclease Xrn1, are detected in *xrn1*Δ mutant cells. **(C)** The 5' NGD-IM derived from the W(UGG)12 reporter assay in the *ski2*Δ background, cleavage site was shifted upstream in *slh1*Δ*ski2*Δ or *uS10-K6/8Rski2*Δ mutant cells. **(D)** The 3' NGD-IM derived from the W(UGG)12 reporter assay in the *xrn1*Δ background, cleavage site was shifted upstream in *slh1*Δ*xrn1*Δ or *uS10-K6/8Rxrn1*Δ mutant cells.
